# Relations of Sanitation to Christianity

**Published:** 1881-05

**Authors:** 


### RELATIONS OF SANITATION TO CHRISTIANITY.

At the Sanitary Convention held under the Management of the Michigan State Board of Health, at Flint, Jan. 25-26, the Rev. C. H. W. Stocking, D. D., gave an address under the above heading, from which the following is an extract: Sanitation, from the first, was religion put into practice. The Saviour's miracles were nearly all for the relief of some form of mental or physical suffering Christianity has ever made sanitation one of the first duties of descipleship. Out of this inherent instinct cf religion the hospital was born, and to Christianity alone the world owes that particular form of organized benevolence.

The church looks upon marriage not merely as a civil contract, but as a divine ordinance, and to be logical, she is bound to teach that the physical and spiritual welfare of the individual and society is laid in Christian Wedlock. Marriages that are likely to perpetrate a feeble, diseased, and vicious stock, are sins against the individual body, the State, and Christianity itself; for the laws of nature are the laws of God also.

Care should be given to the physical education of our children. Many children are sacrificed to rich food, unreasonable hours, fashionable dress, and evening parties. The nursery and the open air, simple food, unfettered limbs in unembroidered garments, romp and play, sleep at the bird's own hour, will prevent many children from drifting into the great ocean of eternity.

When American wives and mothers, sisters and daughters, shall reach the standard of womanhood as given by Solomon in the thirty-first chapter of Proverbs, there will be more intelligent culinary supervision, mutual helpfulness, cheerful economy, and material thrift. There is no part of the great field of humanity and philanthropy where religion is not bound to go with her gentle ministries, and whoever neglects or wars against the body is her enemy. She has a rod to smite every oppressor—the landlord whose tenement house is a death-trap, the rum-seller who steals the frugal gains of the laborer, the ship-master who packs his hold with living freight, and tosses to them starvation fare, the manufacturer who holds in bondage the children who ought to be playing at their mothers knee, or the adulterator of food and drink, whose lying lables cover so many commercial deceits. She condemns him who drugs his senses with alcohol or tobacco, or prostitutes his body to lust, or who shortens his life by inordinate pleasure, needless exposure or overwork, and mad haste for wealth; and may she soon be fearless enough to lift up her voice from all her pulpits against the great host of murderers who in every rank and stations of life, in the church and out of it, are engaged in a cowardly slaughter of the unborn innocents. All readers of current religious events know what she is doing in her great missionary and reformatory enterprises. Where the old brewery and the Five Points once stood, now stand union schools, virtuous women, and palatial warehouses.

Hospital societies, lying-in societies, maternity societies, working-women's homes, are scattered all over the land. Medical missions in China and Japan are the work of Christianity alone, and they have opened in a few months a door at which the preachers of the gospel have been knocking almost in vain for many years.—*Canada Health Journal.*

